# From Chemotherapy to Phototherapy – Changing the Therapeutic Action of a Metallo‐Intercalating Ru^II^‐Re^I^ Luminescent System by Switching its Sub‐Cellular Location

**DOI:** 10.1002/chem.202300617

**Published:** 2023-05-02

**Authors:** Hiwa K. Saeed, Paul J. Jarman, Sreejesh Sreedharan, Rachel Mowll, Alexander J. Auty, Adrien A. P. Chauvet, Carl G. W. Smythe, Jorge Bernardino de la Serna, Jim A. Thomas

**Affiliations:** ^1^ Department of Chemistry University of Sheffield Sheffield S3 7HF UK; ^2^ Department of Biomedical Science University of Sheffield Sheffield S10 2TN UK; ^3^ School of Human Science University of Derby Derby DE22 1GB UK; ^4^ Faculty of Medicine National Heart and Lung Institute Imperial College London SW7 2AZ UK; ^5^ Central Laser Facility Rutherford Appleton Laboratory MRC-Research Complex at Harwell Science and Technology Facilities Council Harwell OX11 0FA UK

**Keywords:** metallointercalator, PDT, ruthenium, rhenium, STED

## Abstract

The synthesis of a new heterodinuclear Re^I^Ru^II^ metallointercalator containing Ru^II^(dppz) and Re^I^(dppn) moieties is reported. Cell‐free studies reveal that the complex has similar photophysical properties to its homoleptic M(dppz) analogue and it also binds to DNA with a similar affinity. However, the newly reported complex has very different in‐cell properties to its parent. In complete contrast to the homoleptic system, the Ru^II^(dppz)/Re^I^(dppn) complex is not intrinsically cytotoxic but displays appreciable phototoxic, despite both complexes displaying very similar quantum yields for singlet oxygen sensitization. Optical microscopy suggests that the reason for these contrasting biological effects is that whereas the homoleptic complex localises in the nuclei of cells, the Ru^II^(dppz)/Re^I^(dppn) complex preferentially accumulates in mitochondria. These observations illustrate how even small structural changes in metal based therapeutic leads can modulate their mechanism of action.

## Graphical abstract

**Chemotherapy: off; phototherapy: on**. A small change in the structure of a STED‐compatible Ru^II^‐Re^I^ theranostic complex not only switches its intracellular localisation but also its mode of therapeutic action.

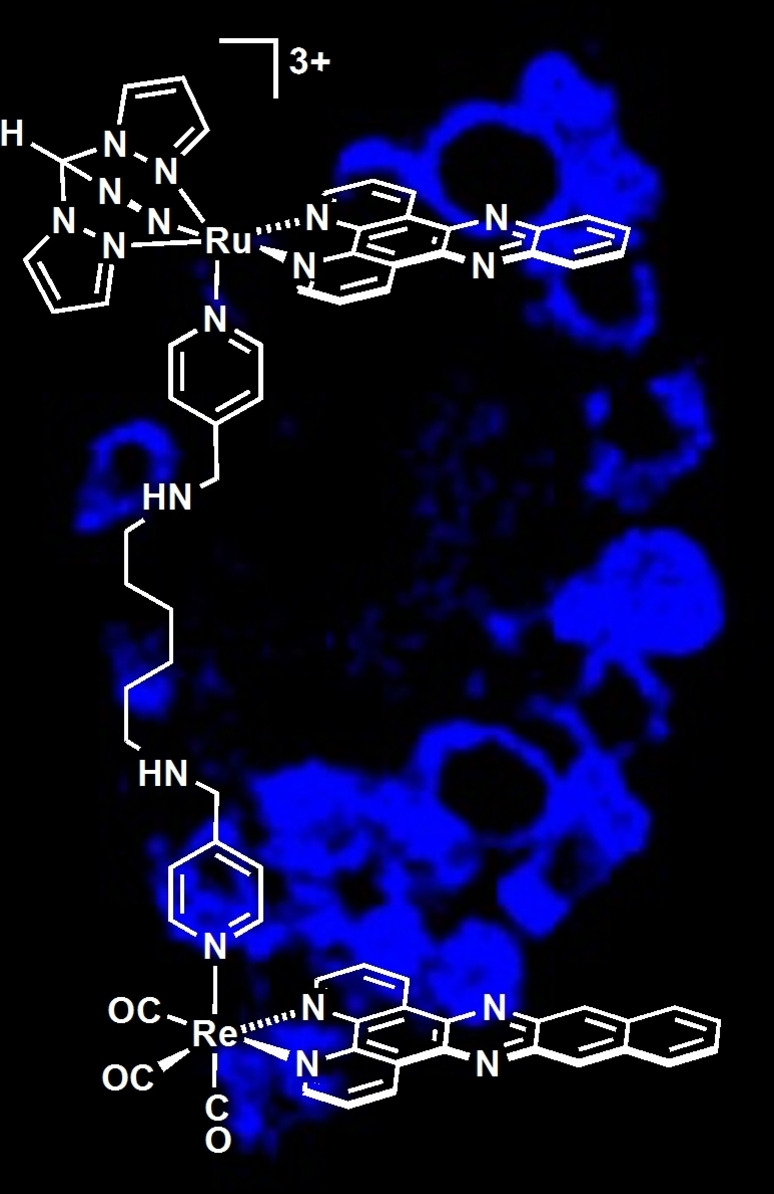

## Introduction

Since the first report of its DNA intercalating properties,^[1]^ the DNA “light‐switch” complex, [Ru(bpy)_2_(dppz)]^2+^, has been the subject of a huge variety of studies[^2^, ^3^, ^4^, ^5^, ^6^] and although it is not cell‐permeant itself,^[7]^ derivatives that are internalized have been developed for a range of applications,[^8^, ^9^, ^10^] including as sensitizers for photodynamic therapy.[^11^, ^12^, ^13^] Analogous systems containing *d* ^6^ metal ions other than Ru^II^ have also been reported, and these complexes can have photophysical and biological properties that are very different to their parent.[^14^, ^15^, ^16^, ^17^] To further modulate and enhance the properties of M(dppz) systems, dinuclear complexes have been reported. The best‐known example of this approach being the threading complexes studied by the Nordén and Lincoln groups, which display extraordinarily high affinities for duplex DNA.[^18^, ^19^, ^20^, ^21^] However, these enantiopure complexes are derived from kinetically inert optical isomers and so their syntheses can be inconvenient.

With the aim of providing a facile route to more structurally complex oligonuclear architectures, the Thomas group has used achiral mononuclear complexes as building blocks in the “modular” synthesis of non‐threading dinuclear metallo‐intercalators.^[22]^ Using this approach metal ions, linkers, and intercalating ligand can all be individually selected, allowing us to explore the properties of dinuclear Ru^II^ systems with two different intercalating ligands^[13]^ or heterodinuclear Ru^II^‐Re^I^ metallo‐intercalators.[^23^, ^24^] We have also found the bridging ligand used to tether the intercalating moieties together can influence the photophysical and biophysical properties of these complexes.[^24^, ^25^]

**Figure 1:**
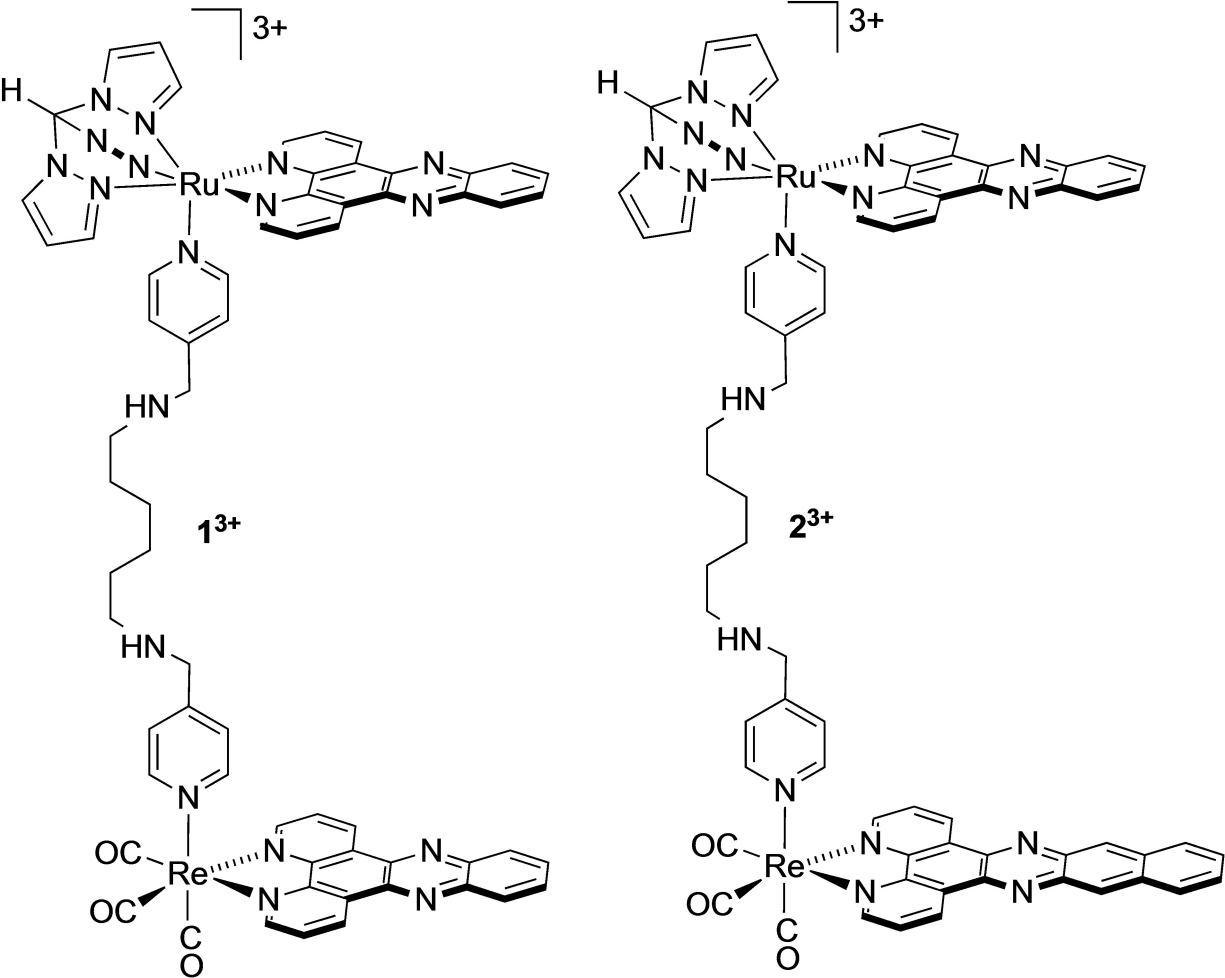
Previously reported complex **1** ^3+^ and newly synthesized complex **2** ^3+^.

In a recent study we reported that, unlike previously described Re^I^(ddpz)‐based complexes, dinuclear Ru^II^/Re^I^ complex, **1** ^3+^ – Figure 1, is not phototoxic. Nevertheless, the complex behaves as a theranostic as it is both conventionally cytotoxic with a potency comparable to cisplatin and a STED nanoscopy probe that selectively localizes in the lysosomes and nuclei of live cells.^[24]^ As we have previously discovered the nature of the intercalating ligand can change both the excited state and biological properties of related homometallic Ru^II^ systems,[^13^, ^26^, ^27^, ^28^] we set out to synthesize and study **2** ^3+^, Figure 1, a heterometallic complex containing two different intercalating ligands, these studies revealed that cytotoxicity, phototoxicity, and live‐cell localization are all profoundly altered by this single change.

## Results and Discussion

This first example of a heteroleptic, heterometallic, bis‐intercalator incorporating both Ru^II^(dppz) and Re^I^(dppn) units was synthesized from the previously reported [Ru(tpm)(L)(dppz)]^2+[29]^ and [ReCl(CO)_3_(dppn)]^+[30]^ using a suitably adapted published procedure to yield **2** ^3+^ as its crude hexafluorophosphate salt. An analytically pure sample of the product was then isolated by repeated anion metathesis – see Supporting Information.

The optical absorption spectrum of [**2**](PF_6_)_3_ shows intense intraligand (IL) π→π* transitions at 235–320 nm, and characteristic bands between 350 and 435 nm, which – in comparison to the free ligands – can be assigned to dppz and dppn‐based IL transitions. Bands observed at 430–500 nm are assigned as MLCT transitions as these are typically observed at these energies for M(dppz) and M(dppn) complexes.

Excitation into the dppz/dppn transitions or the lower energy ^1^MLCT transition results in unstructured luminescence characteristic of the Ru(dπ)→dppz(π*) ^3^MLCT manifold at 657 nm, a red‐shift of 15 nm compared to **1** ^3+^. This observation confirms that the non‐emissive excited state of the Re^I^(dppn) unit is ultimately deactivated by energy transfer to the lower lying Ru‐based ^3^MLCT excited state. The interaction of [**2**]Cl_3_ with calf thymus DNA, CT‐DNA, in aqueous buffer (25 mM NaCl, 5 mmol tris, pH 7.4) was then investigated using UV‐visible absorption and luminescence titrations.

On addition of CT‐DNA both the absorption and emission spectra of the complexes produced changes that are characteristic of DNA binding. Large hypochromicity in both π→π* and MLCT absorption bands as well as accompanying bathochromic shifts are observed; changes that are typical for an intercalative binding mode – Supporting Information Figure S1. Unlike **1** ^3+^, complex **2** ^3+^ is emissive in aqueous solutions; so, although addition of CT‐DNA does result in an increase in its emission, it is not a true “light‐switch” system. Nevertheless, the change in emission, see Supporting Information Figure S2, can be used to construct a saturation binding curve for its interaction with DNA. Fits of this data to the McGhee‐von Hippel model^[31]^ for noncooperative binding led to estimated binding affinity of 1.5×10^5^ M^−1^ (*n*=2 bp). Within error, these figures are identical to those reported for **1** ^3+^.

Having determined that the new complex is luminescent and binds to DNA in cell free conditions with similar affinities to its close analogue its in‐cell properties were then investigated beginning with its cytotoxicity.

**Figure 2:**
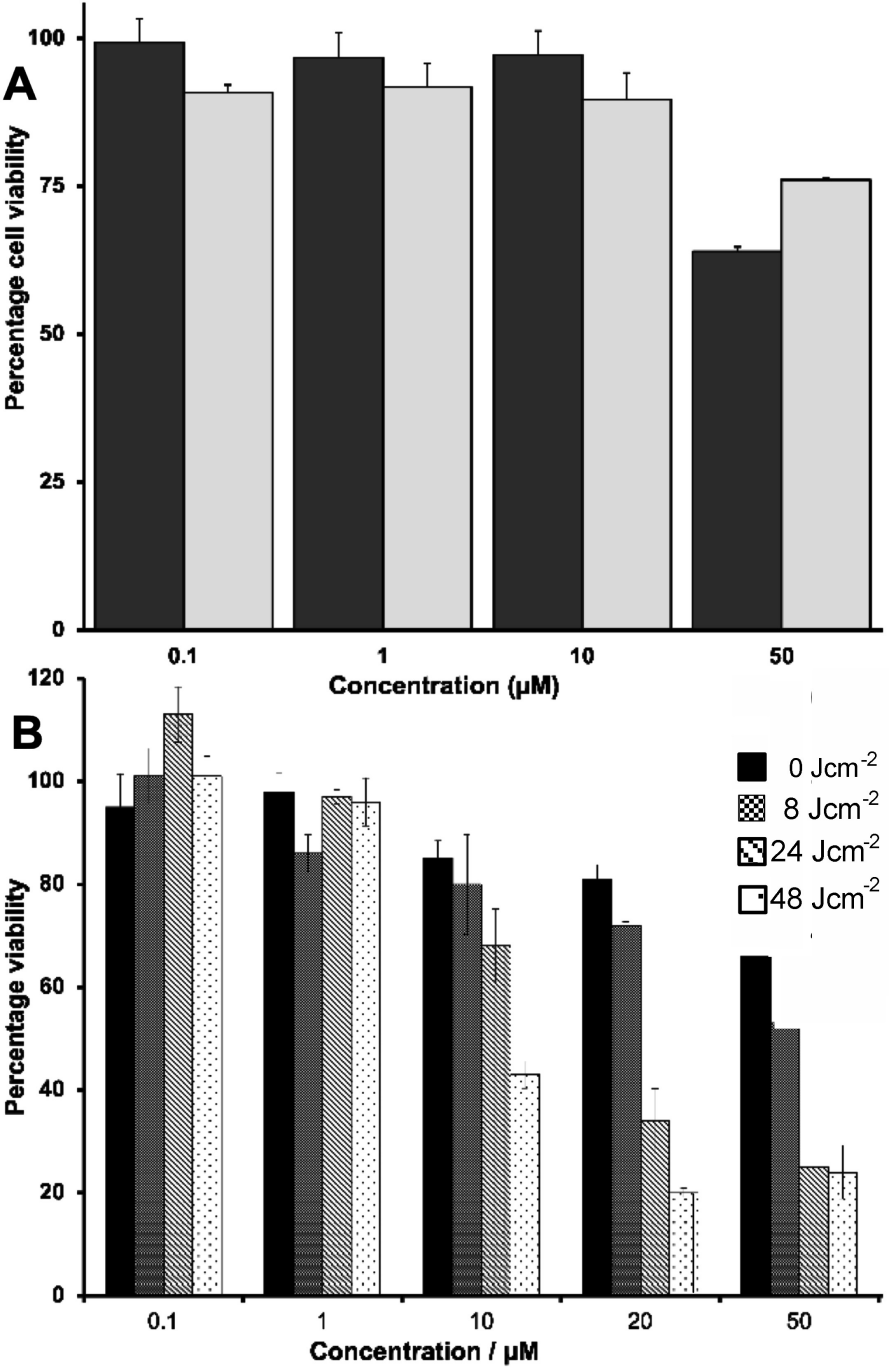
A. IC_50_ values for A2780 (black) and resistant A2780cis (grey) ovarian cancer cells after 48 h exposure to complex **2** ^3+^ as chloride salts. B. A2780 cell viability on exposure to different concentrations of complex **2** ^3+^ and irradiation with broad‐spectrum light at fluences of 0, 8, 24 and 48 J cm^−2^.

This was determined using a range of concentrations (0.1–200 μM) to obtain a 48‐hour IC_50_ value against the ovarian cancer A2780 cell line and its cisplatin resistant variant A2780cis – Figure 2(A). it was found that **2** ^3+^ displays low cytotoxicity against A2780 (IC_50_>50 μM), making it considerably less potent than cisplatin in the same conditions (IC_50_=2 μM). More surprisingly this also means the complex is much less cytotoxic than **1** ^3+^, which displays a IC_50_ of 11 μM in the same conditions.^[24]^ Moreover, although cisplatin displays an expected drop in its activity against the resistant A2780cis line (IC_50_=22 μM), **2** ^3+^ again displays low activity against this line. Again, this contrasts with the properties of **1** ^3+^ against this line (IC_50_=21 μM). As we,[^13^, ^27^] and others,[^32^, ^33^] have demonstrated that M(dppn) complexes often function as singlet oxygen sensitizers for photodynamic therapy, PDT, we then investigated the phototoxicity of the new complex. Using previously published methods,^[34]^ the human ovarian cancer A2780 cells were treated with **2** ^3+^ at concentrations between 0.1 and 200 μM and exposed to broad‐spectrum light at fluences of 0, 8, 24 and 48 J cm^−2^, respectively. The IC_50_ for each of these irradiation conditions was then calculated using MTT assays –Figure 2(B).

Although irradiation of **1** ^3+^ produced no observable phototoxicity, experiments with **2** ^3+^ show that at treatment concentrations of 10 μM and higher a pronounced phototoxic response is observed causing marked decreases in cell viability. The phototoxic index (PI) in the conditions was calculated to be >28, with a fluence of 48 J cm^−2^ resulting in a cytotoxicity (<4 μM) that is – within error – comparable to cisplatin.

Although this potency and its associated PI is appreciably lower than some recently reported systems, the shift from classical chemotoxicity to a significant phototoxic effect is intriguing – particularly given the very close structural similarity between **1** ^3+^ and **2** ^3+^. Therefore, to explore this question in more detail, the photophysical properties of the new complex were further investigated. As M(dppn) systems[^27^, ^35^, ^36^] can be particularly efficient singlet oxygen sensitizers, the ability of **2** ^3+^ to photogenerate ^1^O_2_ was assessed and compared to **1** ^3+^.

Sensitization properties were quantified by the direct measurement of O_2_(^1^Δ_g_)→^3^O_2_ phosphorescence at 1270 nm – Supporting Information, Figure S3. This procedure yielded estimated efficiencies of 28 % (**1** ^3+^) and 34 % (**2** ^3+^), respectively. Although these data suggest **2** ^3+^ is a slightly better sensitizer than **1** ^3+^ they do not seem sufficient to explain the large differences in phototoxicity between the two complexes in themselves. Hence, as the substitution of Re^I^(dppz) with a Re^I^(dppn) moiety had unexpected effects on both the phototoxcity and cytotoxicity of these systems, we compared the cell localization properties of **1** ^3+^ and **2** ^3+^ in more detail.

Previous studies on **1** ^3+^ showed that at low concentrations this complex localizes within lysosomes, but at concentrations above its IC_50_ it moves into the nucleus, and it is the phenomenon that is responsible for the cytotoxicity of **1** ^3+^.^[24]^ However, complex **2** ^3+^ displayed strikingly different behaviour.

**Figure 3:**
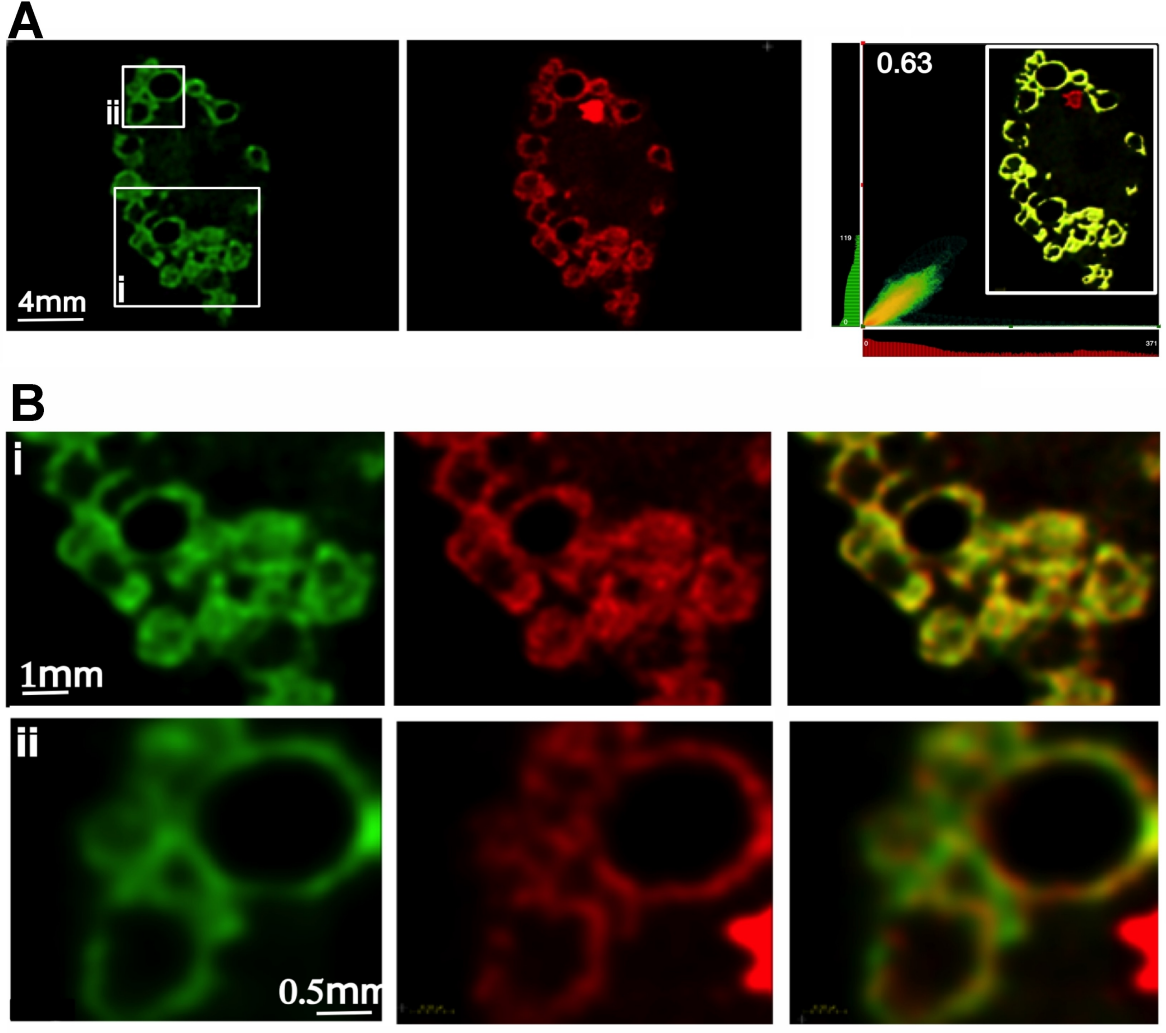
A) Deconvoluted Hyvolution microscopy images of MCF7 cells co‐stained with a commercial mitotracker stain (left) and **2** ^3+^ (center). 2‐D colocalization map (right). B) Details of images shown in boxes (i) and (ii) mitotracker stain (left) and **2** ^3+^ (center), superimposed images (right).

The intracellular localization of **2** ^3+^ was first investigated through enhanced‐resolution deconvoluted laser scanning confocal microscopy (d‐LSCM) technique.^[37]^ The images of cells treated with **2** ^3+^ displayed punctate staining within the cytoplasm, but no nuclear staining. The distinctive pattern is indicative of mitochondrial, rather than lysosomal, staining. This supposition was confirmed by co‐staining experiments with a commercial mitotracker probe which resulted in a Pearson colocalization coefficient of 0.63 with Mitotracker – Figure 3.

**Figure 4:**
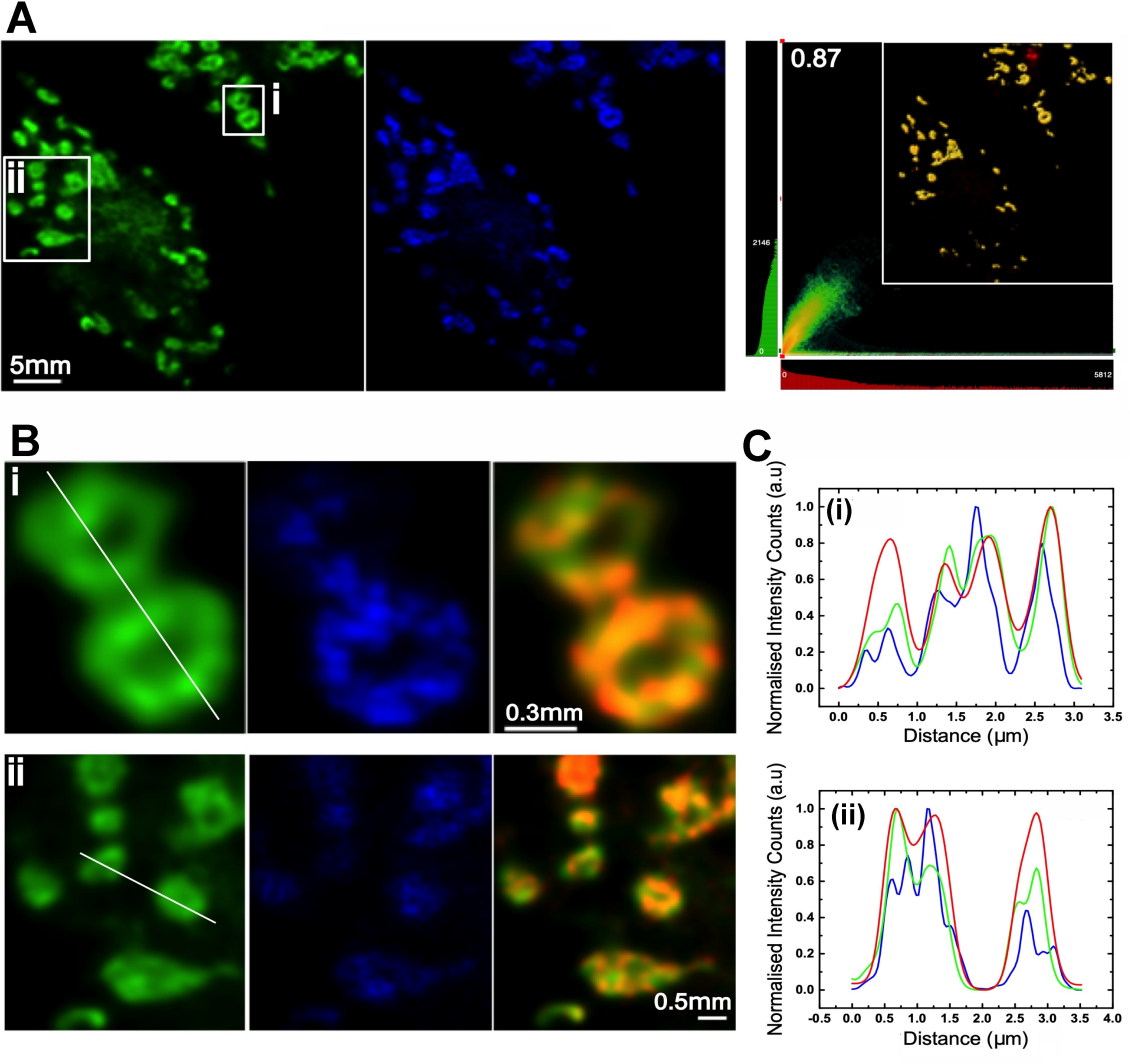
Live cell 3D‐STED images in MCF7 cells of **2** ^3+^. A) Left: Deconvoluted microscopy (Hyvolution) images of Mitotracker; center: Deconvoluted 3D STED image of the same region using the emission of **2** ^3+^; right: Colocalization 2D map showing 87 % colocalization of **2** ^3+^ in mitochodria. B) Magnification of regions in box i (top) and ii (bottom) of A showing (left to right) Hyvolution images of Mitotracker, 3D STED images of of **2** ^3+^, and combined images C. Intensity maps across lines in B (i) and (ii) comparing resolutions of Mitotracker (green), and **2** ^3+^ (red) using deconvoluted Hyvolution microscopy with 3D‐STED imaging of **2** ^3+^ (blue).

Despite its phototoxicity, the luminescent properties of **2** ^3+^ are eminently suited to STED nanoscopy in which a 775 nm depletion beam into the low energy edge of the ^3^MLCT band was employed, allowing deconvoluted STED images to be acquired at sub‐diffraction limited resolutions. A close inspection of the 3D‐STED images sheds more light on live‐cell localization, indicating that complex **2** ^3+^ does not bind mitochondrial DNA but is almost exclusively found within the membrane structures of the mitochondria – Figure 4. The high‐resolution STED images lead to an even higher Pearson colocalization coefficient (0.87) with mitotracker. This observation is not unexpected as a number of studies, including work from our own labs, have shown that cationic metal complexes frequently accumulate within mitochondria; however, the nanoscopy experiments also cast light onto the differing therapeutic function of **1** ^3+^ and **2** ^3+^.

As outlined above, although the addition of the dppn ligand to **2** ^3+^ does slightly increase its ability to function as a singlet oxygen sensitizer compared to **1** ^3+^, the biggest difference between the two complexes is in their sites of cellular accumulation. The localization of cell‐permeant dyes is highly sensitive to small changes in properties such as charge, p*K*_a_, and the balance of lipophilicity/hydrophilicity/amphiphilicity^[38]^ and in this context it is well established that amphiphilic cations like metal complexes most often accumulate in mitochondrial membranes,^[39]^ whereas nuclear targeting is accomplished by more hydrophilic cations.[^13^, ^40^, ^41^] These observations indicate that the substitution of a Re^I^(dppz) moiety found in **1** ^3+^ with a Re^I^(dppn) unit sufficiently increases the amphiphilicity of **2** ^3+^ to switch its localization site. It is this effect that is key to explaining the difference in cytotoxicity and phototoxicity between **1** ^3+^ and **2** ^3+^.

Although both **1** ^3+^ and **2** ^3+^ show appreciable DNA binding affinity in cell‐free conditions and are likely they will bind to an extended sequence in comparison to a mononuclear complex,^[42]^ only **1** ^3+^ localizes in the nucleus. Thus, it is the only complex of the pair that can bind cellular DNA and function as a classical genotoxin. By the same measure, the fact that **2** ^3+^ preferentially accumulates in mitochondria is much more important than any slight increase in its singlet oxygen generating capabilities.

Due to its short cellular lifetime, the diffusion distance of singlet oxygen within cells is limited and although there is some debate on exact figures – estimates ranging from 20 to ∼150 nm – these distances are short enough that PDT effects are only optimized when ^1^O_2_ is generated close to a particularly susceptible cellular target.[^43^, ^44^, ^45^, ^46^, ^47^] In this context, many studies have shown that mitochondria are the critical targets for PDT,[^48^, ^49^] with increased localization of sensitizers within mitochondria, and particularly mitochondrial membranes, resulting in greatly enhanced phototoxicities.[^50^, ^51^, ^52^, ^53^, ^54^] It seems the increased accumulation within mitochondria observed for **2** ^3+^ is responsible for the acquisition of its properties as a PDT sensitizer.

## Conclusions

In conclusion, our studies have shown that switching a single coordinated dppz ligand for a dppn ligand – an addition of only one aromatic ring – leads a dinuclear metallointercalator with a radically different therapeutic function. The change from chemotoxicity to phototoxicity is attributed to the redirection of the new complex away from the nucleus to mitochondria. This study underlines how the modular construction of cell‐permeant, oligonuclear photoactivated metal complexes facilitates their potential as “dial‐in” systems with selectable biological function. As illustrated in this report, the identification of a specific therapeutic action is greatly facilitated by the multimodal imaging properties of these complexes. These properties will be exploited in the development of future probes, therapeutics, and theranostics.

## Experimental Section

**Syntheses**: N,N’‐bis(4‐pyridylmethyl)‐1,6‐hexanediamine (L1), complex **[1]**Cl_3_, [(tpm)Ru(dppz)(L1)]Cl_2_ and **[**Re(CO)_3_Cl(dppn)] were synthesized as previously reported.[^14^, ^24^, ^55^, ^56^]

**[2]Cl_3_**: [Re(CO)_3_Cldppn] (83.5 mg, 0.131 mmol) and AgCF_3_SO_3_ (2.1 eq, 47 mg, 0.182 mmol) were placed in ethanol (50 mL) and heated to reflux overnight. The solution was allowed to cool and filtered through celite to remove the AgCl precipitate. The yellow‐coloured filtrate was returned to the reaction vessel. [(tpm)Ru(dppz)(L1)]Cl_2_ (40 mg, 0.041 mmol) was added and the solution was refluxed overnight again. The solution was allowed to cool to room temperature and then evaporated to obtain a dark red‐brown precipitate. The product was then purified by anion metathesis (36 %); 1H NMR (500 MHz, MeOD): *δ*_H_=9.93 (d, *J*=8 Hz, 2H), 9.89 (d, *J*=8 Hz, 2H), 9.18 (t, *J*=5.5 Hz, 4H), 8.88 (d, *J*=2.5 Hz,4H), 8.61 (m, 4H), 8.33 (d, *J*=2.8 Hz, 4H), 8.20–8.13 (m, 4H), 7.59 (m, 4H), 7.30 (dd, *J*=8.2, 5.4 Hz, 4H), 7.31 (d, *J*=5.9 Hz, 4H), 6.95 (m, 2H), 6.71 (t, *J*=2.5 Hz, 2H), 6.29 (t, *J*=2.5 Hz, 2H), 4.59 (s, 4H), 3.88 (m, 4H), 2.68 (m, 4H), 1.30 (m, 4H); m/z (ES‐MS) 499 (100 %, [M‐3PF_6_]^3+^). Elemental analysis of chloride calcd. (%) for C_71_H_58_Cl_3_N_18_O_3_ReRu.H_2_O: expected C 52.54, H 3.72, N 15.53; found: C 52.44, H 3.91, N 15.51.

## Conflict of interest

The authors declare no conflict of interest.

1

## Supporting information

As a service to our authors and readers, this journal provides supporting information supplied by the authors. Such materials are peer reviewed and may be re‐organized for online delivery, but are not copy‐edited or typeset. Technical support issues arising from supporting information (other than missing files) should be addressed to the authors.

Supporting Information

## Data Availability

The data that support the findings of this study are available from the corresponding author upon reasonable request.
