# Supplementary material for: From Chemotherapy to Phototherapy – Changing the Therapeutic Action of a Metallo‐Intercalating RuII‐ReI Luminescent System by Switching its Sub‐Cellular Location
Source: Chemistry. 2023 May 2;29(34):e202300617. doi: 10.1002/chem.202300617 (PMC10946911; doi:10.1002/chem.202300617)

Chemistry–A European Journal

Supporting Information

From Chemotherapy to Phototherapy – Changing the Therapeutic Action of a Metallo-Intercalating Ru^{II}-Re^{I} Luminescent System by Switching its Sub-Cellular Location

Hiwa K. Saeed, Paul J. Jarman, Sreejesh Sreedharan, Rachel Mowll, Alexander J. Auty, Adrien A. P. Chauvet, Carl G. W. Smythe, Jorge Bernardino de la Serna,* and Jim A. Thomas*

Cell studies

MCF7 cells (HTB-22) were obtained from The American Tissue Culture Collection (ATCC).

Photocytotoxicity (phototoxicity)

Light Irradiation Source Apparatus (LISA)

The apparatus used to irradiate the samples was a custom-made device featuring a broadband illumination source fully contained in an empty computer base unit, referred to as the Light Irradiation Source Apparatus (LISA). The technical specifications of the bulb contained within are as follows (**Table S1**).

Product Code	871691
International Model Number	HC01080i
Description	CFL 80W E40 Integrated
Watts	80W
Cap	E40
Operating Hours	15000
Color Temp	4000K
Lumens	5400 lm
Dimming	No
Dimensions (length x diameter)	256mm x 80mm

Table S1. Specification of the bulb contained within the irradiation apparatus

IC_{50} determination by MTT assay

Cells were maintained in RPMI 1640 medium supplemented with 10% FBS, 100 mg ml^{-1} streptomycin, 100 units ml^{-1} penicillin, and 2 mM glutamine at 37°C in a humidified atmosphere containing 5% CO_2. Experimental cultures were grown on 48 well plates at a seeding density of 5 x 10^4 cells per well and incubated for 24 h. The cells were then treated with complex (solubilised in and maintained at 10% PBS/H_2O: 90% medium throughout all solutions) of a 1-200 μM concentration range, in triplicate, and incubated for 24 h. All complex solution (and control medium) was removed from the cells and replaced with regular growth medium 30 min prior to irradiation. Of the four prepared well plates, one remained in the incubator whilst the other three were exposed to the LISA for the duration of 5, 15 or 30 min (corresponding to light doses of 8, 24 or 48 J cm^{-2}) before being incubated for a further 24 h after culmination of light treatment. All medium was then removed and cells incubated with MTT (0.5 mg ml^{-1} dissolved in PBS) for 40 min. The MTT was removed and formazan product eluted using 120 μl/well acidified isopropanol, 100 μl of which was transferred to a 96 well plate for the absorbance to be quantified by spectrophotometer (595 nm, referenced at 640 nm). An average absorbance for each concentration was calculated and cell viability was determined as a percentage of the untreated negative control wells (10% PBS/H_2O: 90% medium, average of triplicate). Data were plotted in a graph of concentration against cell viability to produce a curve from which the IC_{50} value (half maximal inhibitory concentration) could be derived by interpolation.

Microscopy details

Sample preparation

Live cells Hyvolution imaging, STED and 3D STED imaging

MCF7 cells were incubated in micro slide 8 well is stained with **2^{3+}** (6 μM) and with Mito Tracker Deep Red (500 nM) and live cell imaging was carried out. Before staining the cells were washed regularly three times with RPMI culture media and PBS (two to three times). STED, 3D STED and Hyvolution (Deconvoluted confocal) was carried out in real time. For Hyvolution, **2^{3+}** was excited at 470 nm and the emission was collected between 600 – 650 nm and the Mitotracker Deep Red was excited at 644 nm and the emission was collected in the Alexa fluor channel (> 650 nm). For STED and 3D STED experiments **2^{3+}** was excited at 470 nm, STED depletion was carried out at 775 nm (which depletes **2^{3+}**), the emission was then collected between 600 – 650 nm for **2^{3+}**. Mitotracker deep red was excited at 644 nm, STED depletion was carried out at 660 nm (which depletes Mitotracker deep red) and the emission was collected in the Alexa fluor channel (> 650 nm). Images of live MCF7 cells were taken at 37°C and 5% CO_2. The dye was excited at 470 nm, and STED depleted at 775 nm (for **2^{3+}**) and 660 nm (for Mitotracker deep red); the emission was collected at the wavelength mentioned above and a gating between 2 and 6.5 ns was used.

Details of Microscopy methods

HyVolution, deconvoluted Laser scanning confocal microscopy (d-LSCM)

This methodology developed by Leica microsystems utilises for the image acquisition a reduced size of pinhole right in front of the HyD detectors, and then employs a deconvolution software developed by SVI (Hyugens) in Netherlands. The deconvolution software uses the raw data information from the Leica acquisition files for an optimal deconvolution. This software can use either a theoretical PSF or a calculated one. The single plane images were deconvolved with a theoretical calculated PSF provided by the software and by an experimentally calculated PSF from fluorescent beads, in both case no apparent difference was observed.

STED nanoscopy

STED images were taken in a commercial LEICA SP8 3X gSTED SMD confocal microscope (Leica Microsystems, Manheim, Germany). The microscope is equipped with 3 depletion lines and it is also equipped with a 3D STED additional vortex to obtain higher spatially resolved images in XY and Z. The excitation laser beam consisted of a pulsed (80MHz) super-continuum white light laser (WLL). For a cleaner emission the excitation lines had a clean-up notch filter (NF) in the optical pathway. The gSTED imaging was taken with a continuous wavelength 660 nm nm depletion laser, and the pulsed STED images with a pulsed 775 nm Laser, again in every case the respective NF were in place. The best results were obtained with the pulsed 775 nm laser. The objective employed was a Leica 100x/1.4 NA oil objective. The pinhole was set at one Airy unit. The gated HyD detectors were set with the gated option on and the temporal gated selected was from 2 to 6.5ns when depleting at 660nm. For the 3D STED images the depletion lasers were split in two, the second vortex was set at 50%.

Data processing: Image analysis and deconvolution.

gSTED nanoscopy resolved the localisation and of the mitochondria and Lysosomes with high resolution. To enhance the signal to noise ratio obtained when employing either a continuous wavelength or a pulsed depletion laser deconvolution was applied. Deconvolution was carried out with the software package Huygens (SVI, Netherlands). To quantify the background level of noise we used either an automated quantification provided by the software or a manual by means of computing the averaged background intensity from regions outside the cell. For the deconvolution

we used 40 iterations, a signal to noise ratio of 13, and the classical maximum likelihood estimation method. Colocalisation analysis was performed using the Huygens software package and the Pearson colocalisation coefficient was quantified. The software allows obtaining the 2D colocalisation map of the regions that colocalise. Lines profiles were drawn at regions of interest, the intensity profile over the distance drawn was plotted as normalized intensity employing Origin Pro (OriginLab Corporation, Northampton, MA, U.S.A.). The figures in this paper has been done employing the free open source software Inkscape. The surface rendered images, and intensity images have been processed using Huygens Professional, LAX software (Leica SP8) and the kymographs using a plugin specially designed in Fiji (ImageJ; NIH).

UV-Vis spectra of 50 μM [2]Cl_3 (orange) compared to a equivalent concentration of previously reported [1]Cl_3 (Blue) in 5 mM tris buffer, 25 Mm NaCl, pH 7.4 at 25 °C.

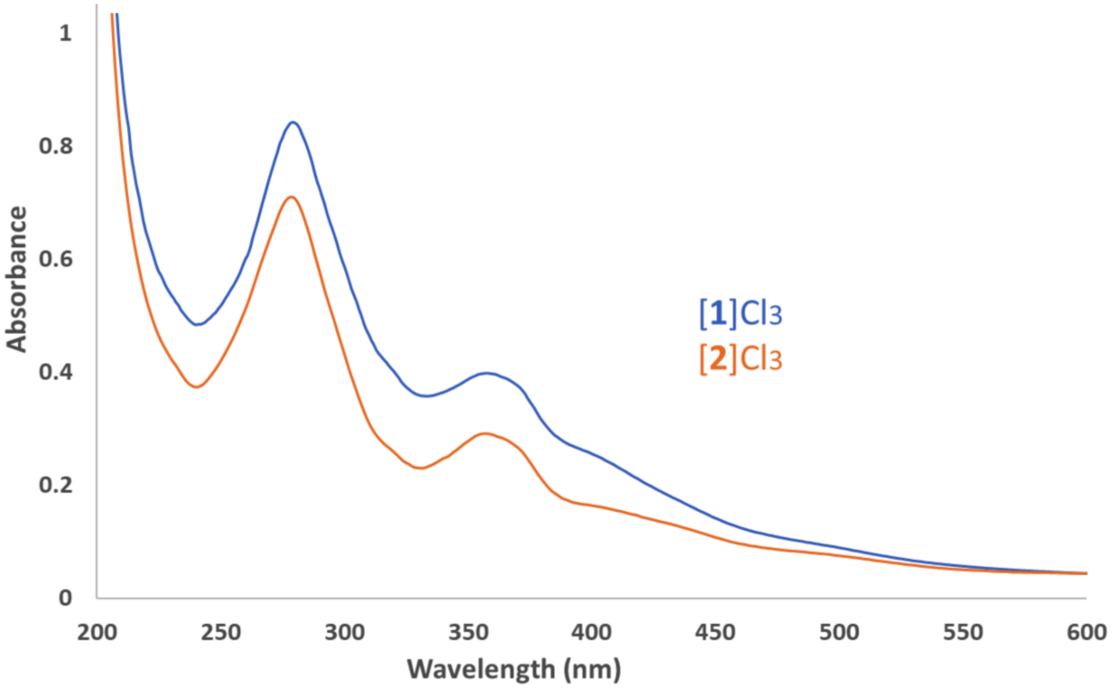

Summary of UV-vis absorbance data for [2]Cl_3

λmax(nm)	10^{-3} ε ($M^{-1}cm^{-1}$)
280	14.00
355	5.95
370	5.8
405	3.0
500	1.80

DNA binding studies

UV-vis titration of complex 2 with ct-DNA

UV-Visible titrations were performed on a thermoregulated Varian-Carey 50 UV-Visible spectrometer at 25°C. 1 ml of buffer was loaded into a 10 mm path length cuvette and allowed to equilibrate inside the spectrometer before a baseline reading was taken. A volume of buffer was removed with a Gilson pipette and replaced with the same volume of a stock solution of complex to give a final concentration of around 50 μM complex inside the cuvette. After equilibration the spectrum was recorded between 200-600 nm. 3 μL of a concentrated stock solution of CT-DNA was added to the cuvette and mixed 10 times with a pipette to ensure homogeneity. The spectrum was recorded after leaving the sample to equilibrate for 5 minutes, checking no bubbles were present. This procedure was continued until the absorbance became constant indicating saturation binding had occurred or the increase of CT-DNA concentration only caused small changes in the absorption spectra.

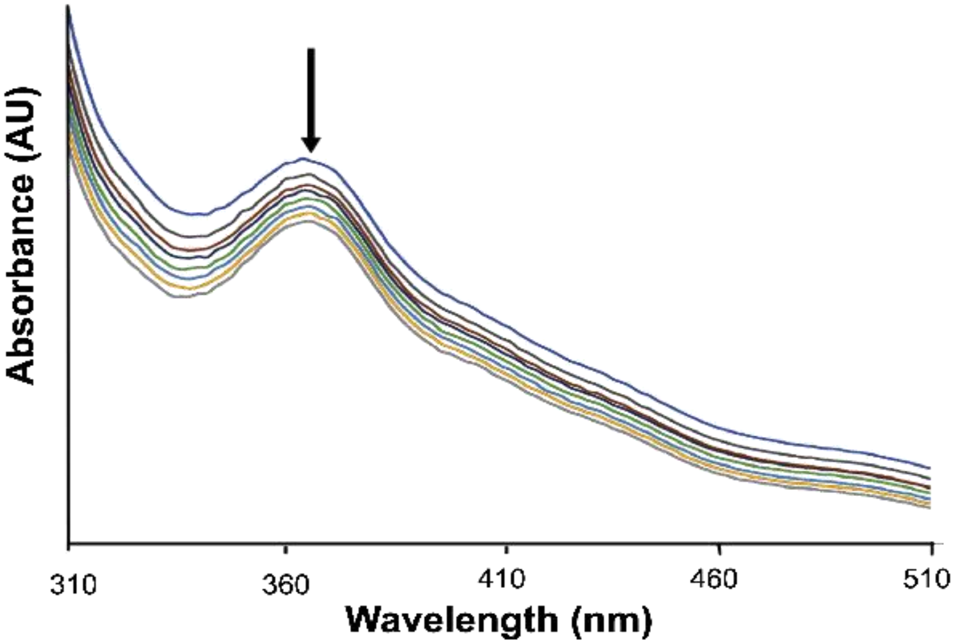

Fig S1. Detaillls of the MLCT region from the UV-Vis titration of 1.01 mM bp^{-1} CT-DNA into a solution of 50 μM [**2**]Cl_3 in 5 mM tris buffer, 25 Mm NaCl, pH 7.4 at 25 °C.

Luminescence titrations

Luminescence titrations were carried out in a thermoregulated Horiba Jobin-Yvon FluoroMax-3 spectrophotometer in a procedure similar to the UV-Visible titrations. 1 mL of buffer was loaded into 10 mm path length luminescence cuvette and allowed to equilibrate inside the spectrophotometer at 25°C before a background reading was taken. A volume of buffer was removed and replaced with the same volume of a stock solution of complex to give a final concentration of around 50 μM complex inside the cuvette. After equilibration, the emission spectrum of the solution was recorded using the excitation wavelength characteristic of the

complex. 3 μL of a concentrated stock solution of CT-DNA was added to the cuvette and mixed 10 times to ensure homogeneity. After leaving the sample to equilibrate for 3 minutes and checking no bubbles were present, the emission spectrum was recorded, showing an enhancement in emission. The procedure was continued until the emission became constant.

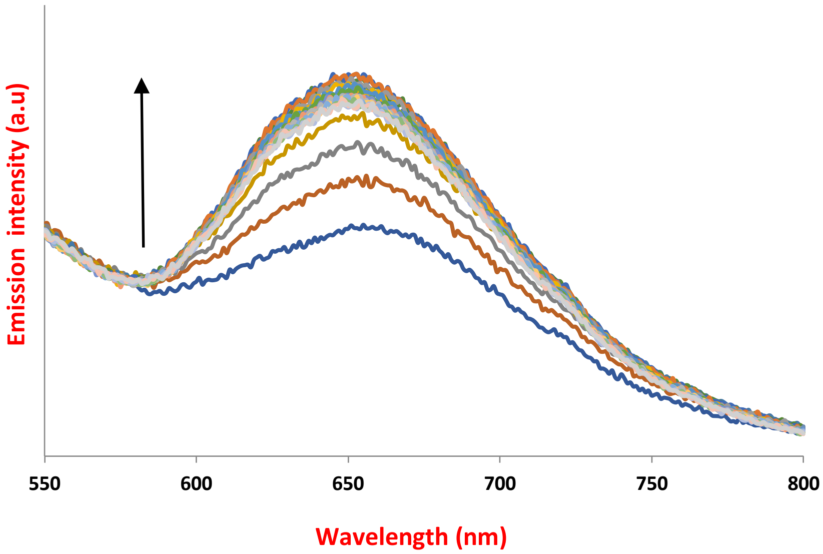

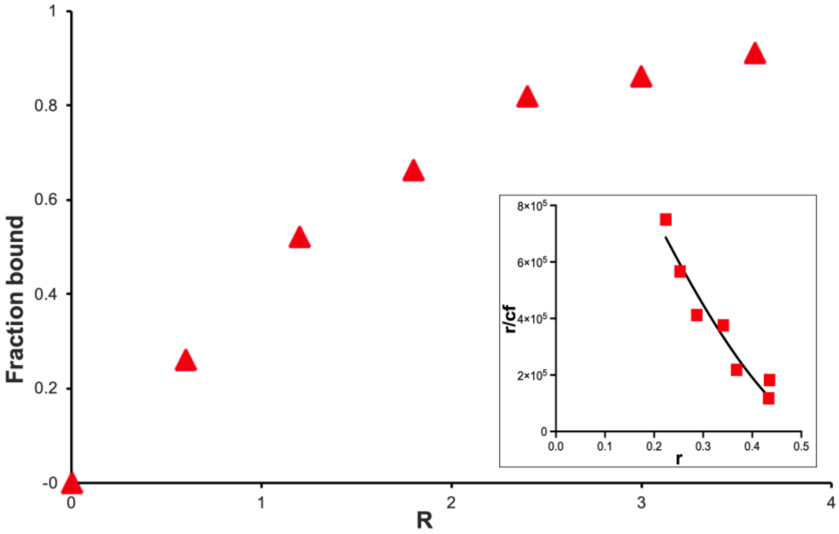

Fig S2. Luminescence titration and binding curve of complex [**2**]Cl_3 in 5 mM tris buffer, with CT-DNA using change in λ_{max} of the emission at 657 nm. Data in the form of a Scatchard plot with a line showing fit to the McGhee-von Hippel model (inset).

Singlet oxygen experiment

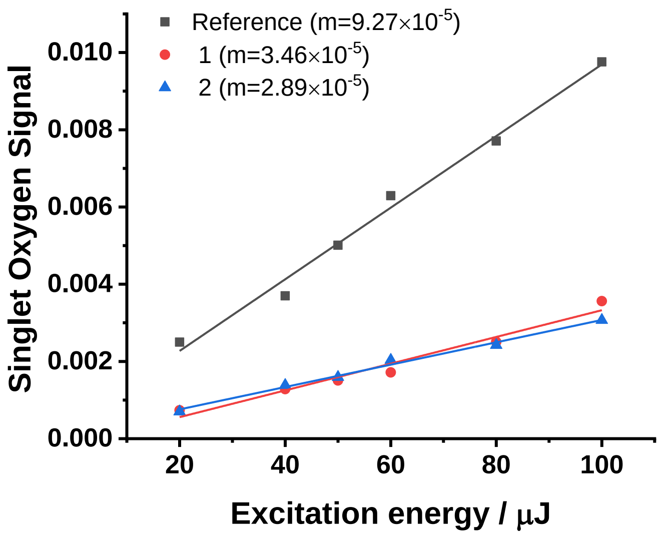

Fig S3. Singlet oxygen quantum yield (φΔ) in dichloromethane was determined employing 355 nm excitation and perinaphthenone as the standard (φΔ perinaphthenone = 95%).[1]
φΔ is calculated by taking the ratio of the gradients (m), of the sample and reference and then applying a correction factor accounting for the difference in optical density at the excitation wavelength (355 nm). $\varphi\Delta_1$ = 0.28 (28%), $\varphi\Delta_2$ = 0.34 (34%).

References

1. N. M. Shavaleev, H. Adams, J. Best, R. Edge, S. Navaratnam, J. A. Weinstein, *Inorg. Chem*. **2006,** 45, 9410-9415.

Appendix: NMR and MS data.

^{1}H NMR spectrum of Complex 2

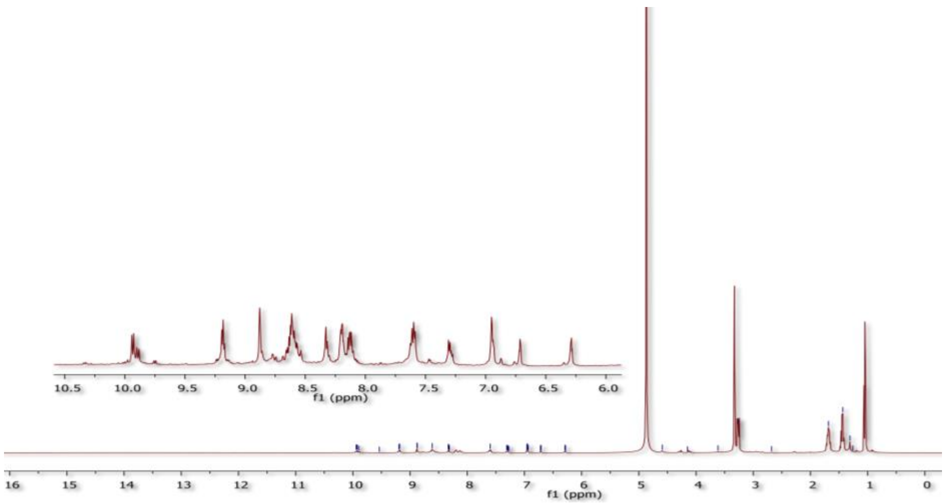

DEPTQ ^{13}C NMR spectrum of complex 2. Due to the poor solubility of the complex, its spectrum is of low quality, but it is included for completeness)

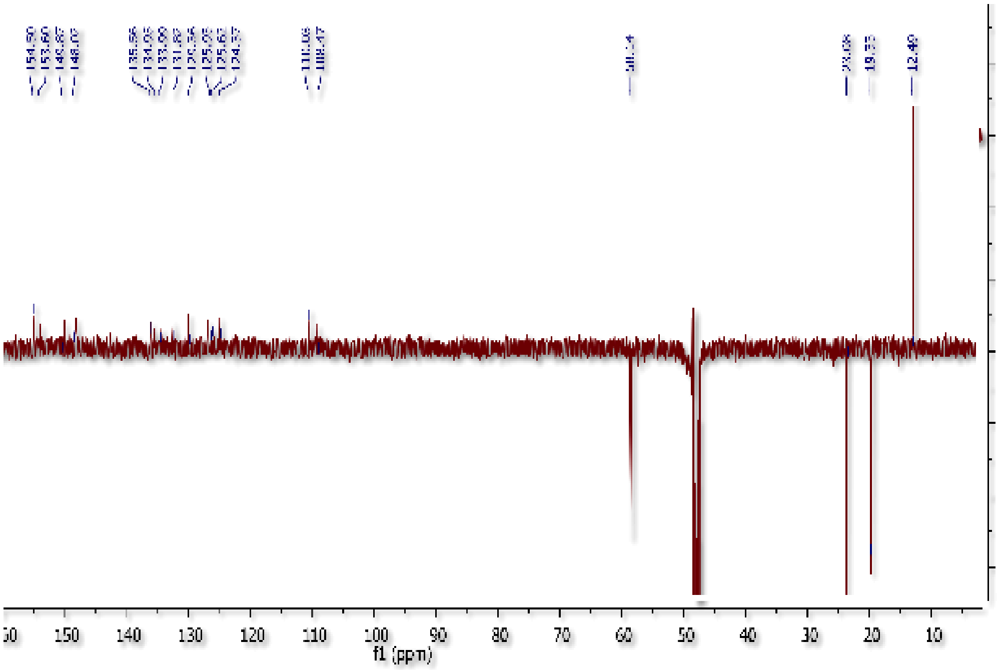

TOF MS ES+ (*m/z*) of complex 2

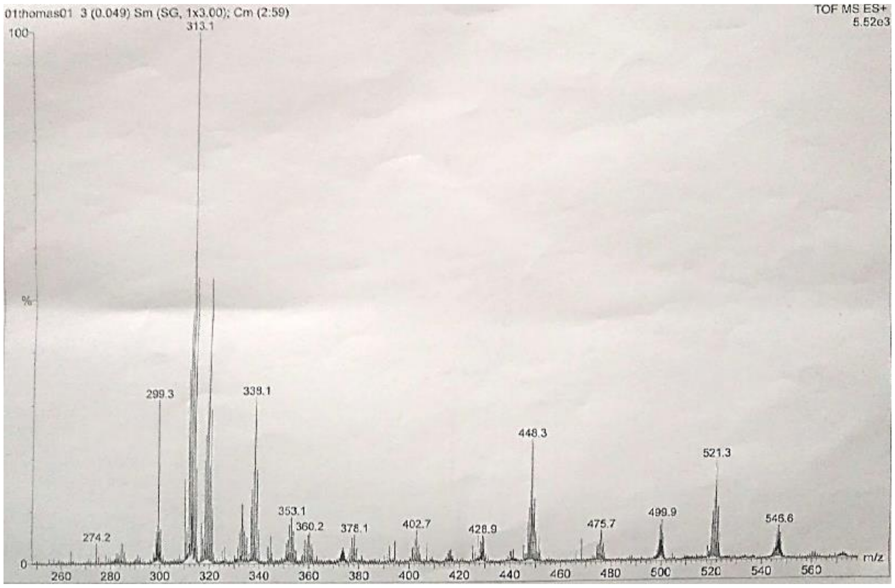

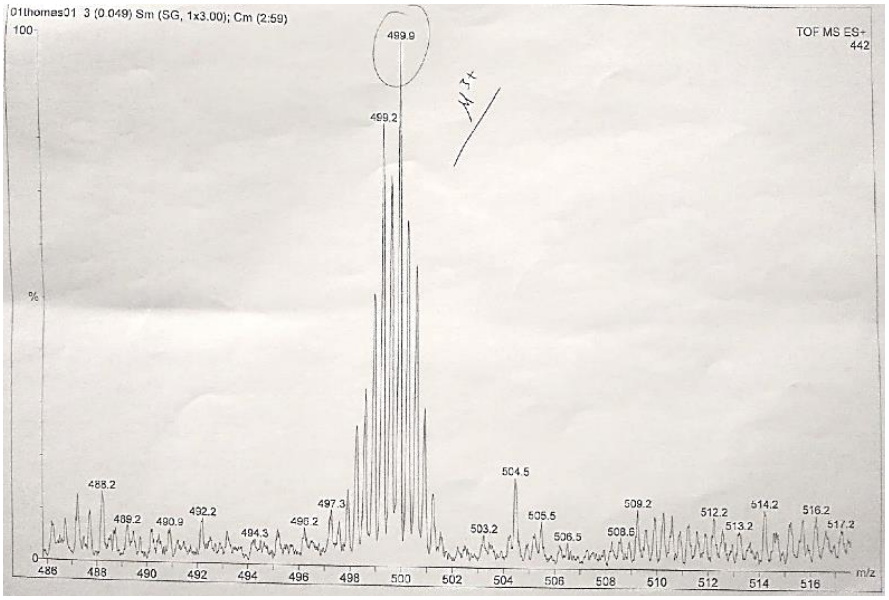

Supplement: Supplementary file 1 — Supporting Information [file CHEM-29-0-s001.pdf]
